# HX008, an anti-PD1 antibody, plus irinotecan as second-line treatment for advanced gastric or gastroesophageal junction cancer: a multicenter, single-arm phase II trial

**DOI:** 10.1136/jitc-2020-001279

**Published:** 2020-10-15

**Authors:** Yan Song, Ning Li, Qun Li, Xinjun Liang, Shu Zhang, Qingxia Fan, Xianli Yin, Zhixiang Zhuang, Yunpeng Liu, Jingdong Zhang, Xiaoge Kou, Haijun Zhong, Xiaofei Wang, Yiwei Dou, Jing Huang

**Affiliations:** 1 Department of Medical Oncology, National Cancer Center/National Clinical Research Center for Cancer/Cancer Hospital, Chinese Academy of Medical Sciences and Peking Union Medical College, Beijing, China; 2 Department of Medical Oncology, Henan Cancer Hospital, The Affiliated Cancer Hospital of Zhengzhou University, Zhengzhou, China; 3 Department of Medical Oncology, Hubei Cancer Hospital, Wuhan, China; 4 Department of Medical Oncology, Shandong Cancer Hospital, Jinan, China; 5 Department of Oncology, The First Affiliated Hospital of Zhengzhou University, Zhengzhou, China; 6 Department of Medical Oncology, Hunan Cancer Hospital, The Affiliated Cancer Hospital of Xiangya School of Medicine, Central South University, Changsha, China; 7 Department of Medical Oncology, The Second Affiliated Hospital of SooChow University, Suzhou, China; 8 Department of Medical Oncology, The First Affiliated Hospital of China Medical University, Shenyang, China; 9 Department of Medical Oncology, Liaoning Cancer Hospital, Cancer Hospital of China Medical University, Shenyang, China; 10 Department of Medical Oncology, The First Affiliated Hospital of Xinxiang Medical University, Xinxiang, China; 11 Department of Medical Oncology, Zhejiang Cancer Hospital, Institute of Cancer and Basic Medicine, Chinese Academy of Sciences, Cancer Hospital of the University of Chinese Academy of Sciences, Hangzhou, China; 12 Taizhou Hanzhong Biomedical Co., Ltd, Jiangsu, China

**Keywords:** gastrointestinal neoplasms, clinical trials, phase II as topic

## Abstract

**Background:**

Irinotecan is used as second-line treatment in advanced gastric or gastroesophageal junction (G/GEJ) cancer. The role of anti-programmed death-1 (PD-1) antibody plus irinotecan, in this setting and population is unclear.

**Methods:**

This multicenter, open-label, single-arm, phase II trial was conducted in 11 Chinese hospitals. Eligible patients had histologically confirmed advanced G/GEJ cancer that refractory to, or intolerant of, first-line chemotherapy with a platinum and/or fluoropyrimidine. Subjects received HX008 200 mg intravenously every 3 weeks plus irinotecan 160 mg/m^2^ intravenously every 2 weeks until disease progression or unacceptable toxicity. The primary end point was objective response rate (ORR) as assessed according to Response Evaluation Criteria In Solid Tumors V.1.1.

**Results:**

Between October 2018 and September 2019, a total of 58 patients with advanced G/GEJ cancer were enrolled in this study. Median follow-up was 10.5 months (range 7.4–18.9) months. Confirmed ORR was observed in 16 patients, for an ORR of 27.6% (95% CI 16.1% to 39.1%); 19 patients experienced stable disease, leading to a disease control rate of 60.3% (95% CI 46.4% to 73.0%). ORR in patients with PD-ligand 1 (L1) positive (Combined Positive Score (CPS) ≥1) and negative (CPS＜1) tumors was 38.5% (5/13) and 37.5% (3/8), respectively. Median duration of response was 8.0 months (range 1.5–12.5), 6 of 16 (37.5%) responses were ongoing. Median progression-free survival (PFS) was 4.2 months (95% CI 2.2 to 5.5). Median overall survival (OS) was not reached (NR) (95% CI 8.7 to NR). Patients with PD-L1 positive tumors tended to have longer OS than those with PD-L1 negative tumors, but the difference was not statistically significant (NR vs 8.7 months, p=0.1858).

The most common treatment-related adverse events of grade 3 or 4 included neutropenia (32.8%), leukopenia (31.0%), anemia (17.2%), decreased appetite (8.6%), vomit (6.9%), nausea (6.9%) and fatigue (5.2%). There were no treatment-related deaths.

**Conclusion:**

The combination of HX008 and irinotecan demonstrated promising activity and manageable safety as second-line treatment in patients with advanced G/GEJ cancer, which warrants further study.

**Trial registration number:**

NCT03704246

## Background

Gastric cancer (GC) is the fifth most common cancer worldwide and the third-leading cause of cancer-related death, and more than half of the total cases occur in Eastern Asia.^1 2^ GC is mostly diagnosed at an advanced stage due to its non-specific symptoms, which is associated with a poor overall survival (OS). The standard of care for first-line treatment of advanced GC is fluoropyrimidine-based and platinum-based chemotherapy, patients with human epidermal growth factor receptor 2-positive tumors should also receive trastuzumab.^3^ In second-line setting, taxane or irinotecan monotherapy, or ramucirumab alone or in combination with paclitaxel is the validated therapeutic options for patients with adequate condition status.^4^ However, the 5-year OS rate of metastatic gastric adenocarcinoma is still estimated around 5%–20%,^5^ underscoring the need for effective therapies with acceptable safety profiles.

Immune checkpoint inhibitors (ICIs) targeting programmed death receptor 1 (PD-1) and PD-ligand 1 (PD-L1) enhance antitumor T-cell activity through inhibition of suppression signals, and have improved OS of patients with various types of cancers, including GC.^6 7^ In ATTRACTION-2 study, nivolumab monotherapy demonstrated a significantly longer OS vs placebo (5.3 vs 4.1 months; HR 0.63; 95% CI 0.51 to 0.78; p＜0.0001), regardless of PD-L1 expression in advanced gastric or gastroesophageal junction (G/GEJ) cancer that refractory to or intolerant of ≥2 prior chemotherapy regimens.^7^ In KEYNOTE-059 study, pembrolizumab monotherapy elicited durable objective responses in 30 of 259 patients (11.6%) who had disease progression after two or more prior chemotherapy regimens in advanced G/GEJ adenocarcinoma, and more durable responses were confirmed in patients with PD-L1 positive tumors.^6^ However, pembrolizumab monotherapy failed to manifest superior survival as compared with paclitaxel in the second-line setting of patients with PD-L1 Combined Positive Score (CPS) ≥1 G/GEJ cancer in KEYNOTE-061 study.^8^ Besides, pembrolizumab monotherapy or combined with chemotherapy as first-line treatment in patients with PD-L1 CPS ≥1 G/GEJ cancer was not superior as compared with chemotherapy in KEYNOTE-062 study.^9^

Combination therapy as second-line treatment might enhance clinical efficacy. Combination of ramucirumab with paclitaxel is the only therapy that has engendered superior OS as compared with paclitaxel monotherapy.^10^ However, ramucirumab has not been approved in China, leaving substantial and urgent unmet medical needs for such patients. Combination with ICIs and chemotherapy has proved to improve OS in several cancer types,^11–15^ mechanistically via synergistic antitumor activity through modulation of the tumor microenvironment.^16–18^ Limited data of combination ICIs and chemotherapy or other antitumor agents as second-line treatment for advanced GC were revealed.

HX008 is a novel highly selective, fully humanized monoclonal antibody that blocks the interaction between PD-1 and its ligands.^19^ The role of anti-PD-1 antibody plus irinotecan in advanced G/GEJ cancer is unclear. In the present study, we conducted a 2-cohort phase 2 trial in which cohort 1 was designed to evaluate the antitumor activity and safety of HX008 plus irinotecan as second-line treatment in patients with advance G/GEJ cancer.

## Methods

### Study design and participants

This phase II clinical study was a multicenter, open-label, non-randomized, 2-cohort, phase 2 trial that conducted across 11 hospitals in China. Eligible patients in cohort 1 were ≥18 and≤75 years of age with histologically confirmed diagnosis of advanced G/GEJ cancer; refractory to, or intolerant of first-line chemotherapy (platinum-containing and/or fluorouracil-based regimen); at least one measurable lesion diagnosed according to the Response Evaluation Criteria In Solid Tumors (RECIST) V.1.1 at baseline; an Eastern Cooperative Oncology Group (ECOG) performance status score of 0 or 1; a life expectancy ≥3 months and adequate organ function. Key exclusion criteria were: active or prior documented autoimmune or inflammatory disease; prior treatment with an agent directed against PD-1, or PD-L1 or CTLA-4; history or current interstitial lung disease or pulmonary fibrosis; symptomatic central nervous system metastases; exposure to any investigational agent within 4 weeks of the first dose of study drug; the adverse reactions of previous treatments failed to recover to National Cancer Institute Common Terminology Criteria for Adverse Events (CTCAE; V.5.0) grade score ≤1.

Written informed consent was obtained from all patients before enrolment.

### Study treatment

All subjects received HX008 combined with irinotecan. HX008 200 mg was administrated intravenously over 60 min every 3 weeks up to 2 years; irinotecan 160 mg/m^2^ was administered intravenously over 60–120 min every 2 weeks. For each patient during the overlapping cycle of HX008 and irinotecan, HX008 was administrated first, followed sequentially by the irinotecan infusion. Treatment was discontinued on disease progression, unaccepted toxicity, physician decision or patient withdrawal of consent, whichever came first. Clinically stable patients with the first radiographic evidence of progressive disease (PD) could remain on treatment at the investigator’s discretion until confirmed PD. Treatment interruptions were permitted for the management of treatment-related AEs.

### End points and assessments

The primary endpoint was objective response rate (ORR) per RECIST V.1.1, which was defined as the percentage of patients with a confirmed complete (CR) or partial response (PR). Secondary endpoints included disease control rate (DCR), duration of response (DOR), PFS, OS and safety. DCR was defined as the percentage of patients with a confirmed CR or PR, or stable disease (SD). DOR was defined as time from initial radiographically CR or PR until disease progression. PFS was defined as the time from the date of registration to disease progression or death from any cause, whichever occurred first. OS was defined as the time from the date of registration to death from any cause.

Tumor imaging was performed by CT scans or MRI at baseline, and every 6 weeks thereafter through the first year, then every 12 weeks. RECIST V.1.1 and immune related Response Criteria (iRECIST) were used for tumor Evaluation. Adverse events (AEs) were recorded during the study period from the initiation of treatment to 30 days after the last dose or the start date of subsequent antitumor therapy followed the last dose, whichever came first. Immune-related AEs should be recorded up to 90 days after the last administration of HX008. AEs were coded using the Medical Dictionary for Regulatory Activities (V.20.0) and graded according to the CTCAE V.5.0.

### PD-L1 detection

For patients with available tumor samples, PD-L1 tumor expression was assessed by immunohistochemistry in archival or newly collected tumor samples at the central laboratory (SAB028, Shuwen Biotech). PD-L1 expression was measured using the CPS, defined as the number of PD-L1-positive cells (tumor cells, lymphocytes, macrophages) as a proportion of the total number of tumor cells multiplied by 100. Tumors was considered to have positive PD-L1 expression when CPS ≥1.

### Statistical methods

In this trial, 55 patients were enrolled to obtain a planned sample size of 50 patients if 10% of enrolled patients would not be treated. This provided approximately 80% power, at a one-sided nominal α=0.05, to detect an ORR of 22% or greater with HX008 plus irinotecan compared with a fixed control rate of 10% (based on historical data) using the exact binomial test. Efficacy endpoints were analyzed in the full analysis set (FAS), defined as patients who received at least one dose of study treatment and for whom any efficacy data on treatment were available. Safety endpoints were assessed in patients who received at least one dose of study treatment. ORR and DCR were assessed based on the point estimate and 95% CI using the Clopper-Pearson exact method. The subjects without mitigation data in FAS would be counted as no remission. PFS, DOR and OS were estimated by Kaplan-Meier method. Statistical analysis was performed using SAS V.9.4 (SAS Institute) statistical software.

## Results

### Patients characteristics

Between October 2018 and September 2019, a total of 58 patients with advanced G/GEJ cancer were enrolled from 11 centers in China. All patients received at least one dose of HX008 combined with irinotecan. Baseline characteristics are listed in table 1. Median (range) age was 60 (26–70) years. Most patients were male (72.4%) and had an ECOG performance status score of 1 (58.6%). Forty patients were advanced GC and 18 patients were advanced gastroesophageal junction cancer. PD-L1 expression was detected in 22 patients with available tumor samples, with 14 patients had PD-L1 positive tumors.

**Table 1:** Baseline characteristics (full analysis set)

	No of patients (N=58)	%
Age (years)		
Median (range）	60 (26–70)	
Sex		
Male	42	72.4
Female	16	27.6
ECOG PS		
0	24	41.4
1	34	58.6
Primary tumor site		
Gastric cancer	40	69.0
Gastroesophageal junction cancer	18	31.0
No of metastatic sites		
1	30	51.7
≥2	28	48.3
Previous tumor surgery		
Yes	28	48.3
No	30	51.7
Previous chemotherapy		
Platinum	54	93.1
Fluoropyrimidine	57	98.3
Taxanes	23	39.7
Liver metastases		
Yes	29	50.0
No	29	50.0
Lung metastases		
Yes	11	19.0
No	47	81.0
Lymph node metastases		
Yes	46	79.3
No	12	20.7

ECOG PS, Eastern Cooperative Oncology Group performance status.

By the data cut-off date of May 11 2020, the median follow-up was 10.5 months (range 7.4–18.9). Six patients (10.3%) continued to receive study treatment. The most common reason for treatment discontinuation was disease progression (39 patients (67.2%)) (online supplemental figure 1). The median duration of exposure to study treatment was 3.5 months (range 0.03–16.3), and the median number of HX008 and irinotecan were 5 (range 1–22) and 7 (range 1–32), respectively.

10.1136/jitc-2020-001279.supp1 Supplementary data

### Antitumor activity

**Figure 1:**
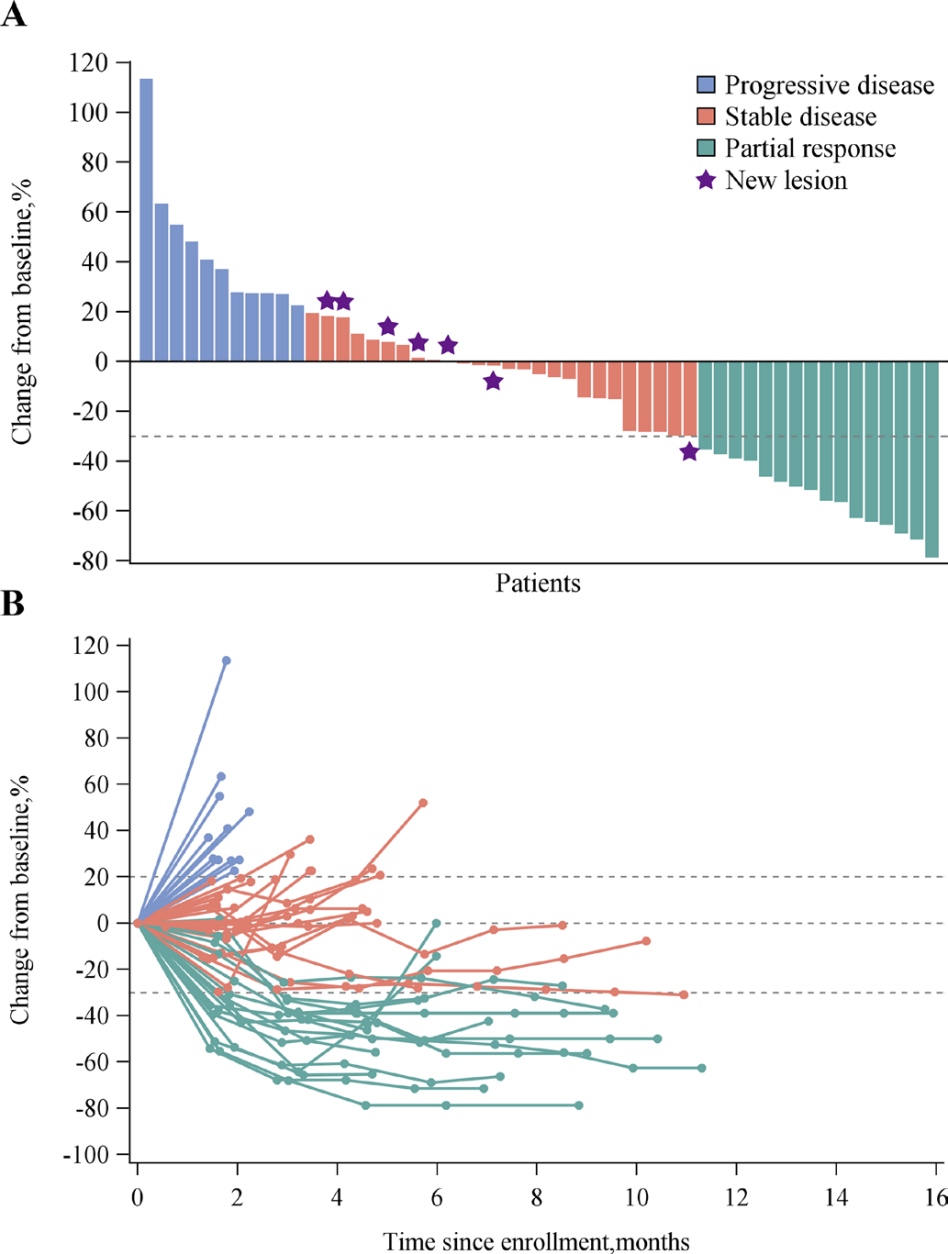
Overall tumor responses of HX008 plus irinotecan as assessed by site investigators. (A) Best change from baseline in the size of target tumor lesion. Color code defines the best of response of target tumor lesion. Seven patients (indicated by star) had a new lesion were evaluated as disease progression. (B) Percent change in the size of target tumor lesion from baseline in each patient.

Tumor evaluations were listed in table 2. Confirmed PR was observed in 16 patients, for a confirmed ORR of 27.6% (95% CI 16.1% to 39.1%); 19 patients experienced SD, leading to a DCR of 60.3% (95% CI 46.4% to 73.0%). ORR and DCR in evaluable patients were 30.2% (95% CI 18.3% to 44.3%) and 66.0% (95% CI 51.7% to 78.5%), respectively. Six of the responses (37.5%) were ongoing at the time of data cut-off. The median DOR was 8.0 months (range 1.5–12.5) and the median time to response was 1.4 months (range 1.3–7.8). Among patients with ≥1 evaluable postbaseline imaging assessment, 32 (60.4%) experienced reduction in measurable tumor size (figure 1A), and decrease in tumor burden was maintained over several assessments (figure 1B).

**Table 2:** Antitumor activity assessed by sites investigators per RECIST V.1.1

Antitumor activity	Total
	N=58
ORR, n (%) (95% CI) *	16 (27.6) (16.7 to 40.9)
DCR, n (%) (95% CI) *	35 (60.3) (46.6 to 73.0)
Best overall response, n (%)	
Complete response	0 (0.0)
Partial response	16 (27.6)
Stable disease	19 (32.8)
Progressive disease	18 (31.0)
Not evaluable	5 (8.6)
Time to response† months, median (range)	1.4 (1.3–7.8)
Duration of response†‡ months, median (range)	8.0 (1.5–12.5)
PFS‡, months, median (95% CI)	4.2 (2.2 to 5.5)
6-month rate (95% CI)	28.3 (16.5 to 41.3)
OS‡, months, median (95% CI)	NR (8.7 to NR)
12 month rate (95% CI)	64.2 (49.4 to 75.7)

*Based on the Clopper-Pearson exact method.

†Evaluated in patients who had a complete or partial response.

‡Estimated using the Kaplan-Meier method.

DCR, disease control rate; NR, not reached; ORR, objective response rate; OS, overall survival; PFS, progression-free survival; RECIST, Response Evaluation Criteria In Solid Tumors.

**Figure 2:**
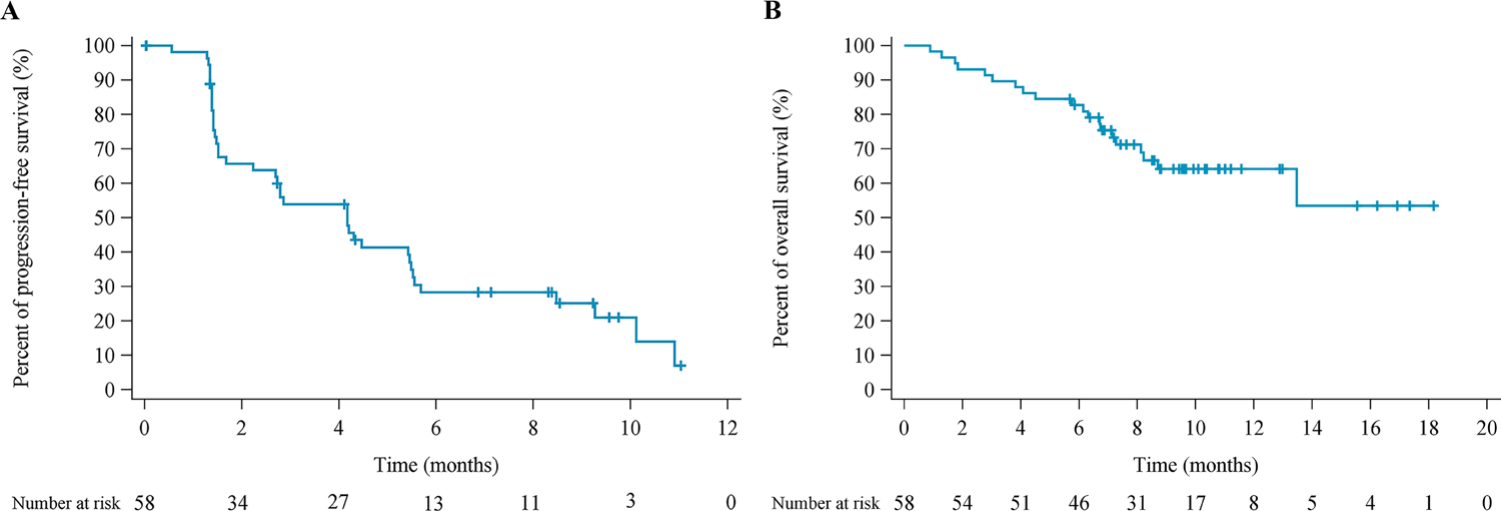
Kaplan-Meier estimates of progression-free survival (A) and overall survival (B).

By data cut-off, 39 patients had disease progression and 20 patients died. Median PFS was 4.2 months (95% CI 2.2 to 5.5), and the estimated 6-month PFS rate was 28.3% (95% CI 16.5% to 41.3%) (table 2 and figure 2A). Median OS was not reached (NR) (95% CI 8.7 to NR) and the estimated 12 month OS rate was 64.2% (95% CI 49.4% to 75.7%) (figure 2B).

PD-L1 expression could be evaluated in 22 patients at baseline, and 14 patients (63.6%) with PD-L1 CPS of 1 or higher. Among patients with evaluable patients, ORR was 38.5% (5/13) with CPS≥1 and 37.5% (3/8) with CPS＜1, DCR was 84.6% (11/13) and 75% (6/8), respectively. Median PFS was 4.3 months with CPS ≥1 and 5.0 months with CPS＜1, respectively. Median OS was NR with CPS ≥1 and 8.7 months with CPS＜1, respectively.

### Safety

Treatment-related AEs (TRAEs) of any grade occurred in all patients (table 3). The most common TRAEs of any grade were leukopenia (81.0%), neutropenia (77.6%), nausea (65.5%), vomit (60.3%), decreased appetite (53.4%), fatigue (50.0%), anemia (48.3%), diarrhea (44.8%), decreased lymphocyte count (24.1%) and weight loss (22.4%). Grade 3 or 4 TRAEs occurred in 37 patients (63.8%); the most frequent (>5%) ones included neutropenia (32.8%), leukopenia (31.0%), anemia (17.2%), decreased appetite (8.6%), vomit (6.9%), nausea (6.9%) and fatigue (5.2%). Serious AEs (SAEs) were reported in 10 patients (17.2%), with the most common being neutropenia (6.9%), anemia (5.2%), decreased appetite (5.2%), diarrhea (3.4%) and vomit (3.4%) (table 3). The most common immune-related AEs were grade 1 or 2, including fatigue (29.3%), proteinuria (15.5%), hypothyroidism (13.8%), diarrhea (12.1%), rash (10.3%) (table 4).

**Table 3:** TRAEs of any grade occurring in ≥10% of patients

Treatment-related AEs* n (%)	Total N=58		
	Any grade, %	Grade 3, %	Grade 4, %
Any adverse event	58 (100)	37 (63.8)	8 (13.8)
Treatment-related SAEs	10 (17.2)	6 (10.3)	3 (5.2)
TRAEs leading to discontinuation	9 (15.5)	4 (6.9)	2 (3.4)
TRAEs leading to dose delay	20 (34.5)	15 (25.9)	2 (3.4)
TRAEs leading to dose reduction of irinotecan	23 (39.7)	16 (27.6)	4 (6.9)
TRAEs (≥10%)			
Hematologic			
Leukopenia	47 (81.0)	18 (31.0)	2 (3.4)
Neutropenia	45 (77.6)	17 (29.3)	7 (12.1)
Anemia	28 (48.3)	10 (17.2)	0
Decreased lymphocyte count	14 (24.1)	2 (3.4)	0
Thrombocytopenia	9 (15.5)	1 (1.7)	0
Non-hematologic			
Nausea	38 (65.5)	4 (6.9)	0
Vomit	35 (60.3)	4 (6.9)	0
Decreased appetite	31 (53.4)	5 (8.6)	0
Fatigue	29 (50.0)	3 (5.2)	0
Diarrhea	26 (44.8)	0	1 (1.7)
Weight loss	13 (22.4)	1 (1.7)	0
Proteinuria	10 (17.2)	0	0
Hypothyroidism	10 (17.2)	0	0
ALT increased	9 (15.5)	0	0
AST increased	9 (15.5)	0	0
Alopecia	9 (15.5)	0	0
Hypoalbuminaemia	8 (13.8)	0	0
Abdominal pain	7 (12.1)	1 (1.7)	0
DBIL increased	7 (12.1)	0	0
Rash	6 (10.3)	0	0

*Attribution of AEs to study treatment was determined by the investigators. Grade 3 and grade 4 are overlapping.

AE, adverse event; ALT, alanine aminotransferase; AST, aspartate aminotransferase; DBIL, direct bilirubin; SAE, serious adverse event; TRAEs, treatment-related adverse events.

**Table 4:** Immune-related adverse events (AEs)

Immune-related AEs n (%)*	Total N=58		
	Any grade, %	Grade 3, %	Grade 4, %
Any adverse event	30 (51.7)	2 (3.4)	0
Fatigue	17 (29.3)	2 (3.4)	0
Proteinuria	9 (15.5)	0	0
Hypothyroidism	8 (13.8)	0	0
Diarrhea	7 (12.1)	0	0
Rash	6 (10.3)	0	0
Pruritus	5 (8.6)	0	0
Myocarditis	1 (1.7)	0	0
Testosterone decrease	1 (1.7)	0	0

*Attribution of AEs to study treatment was determined by the investigators.

Nine patients (15.5%) had AEs that lead to permanent HX008 and/or irinotecan discontinuation, including neutropenia (5.2%), vomit (5.2%), leukopenia (3.4%), fatigue (3.4%) and anemia (1.7%). Dose reduction of irinotecan was performed in 23 (39.7%) out of the 58 patients. The most frequent (>5%) reasons of dose reduction of irinotecan were neutropenia (20.7%), leukopenia (10.3%), nausea (10.3%), vomit (10.3%), decreased appetite (10.3%) and fatigue (8.6%). There were no treatment-related deaths.

## Discussion

Patients with advanced GC whose disease progresses after first-line therapy have limited treatment options. In this multicenter, open-label, phase II trial of patients refractory to, or intolerant of first-line chemotherapy, HX008 plus irinotecan demonstrated promising antitumor activity and manageable toxic effects. To the best of our knowledge, this is the first report to evaluate the efficacy and safety of the combinations of anti-PD-1 monoclonal antibody and chemotherapy as second-line therapy in advanced G/GEJ adenocarcinoma.

Although irinotecan has been standard second-line treatment in patients with advanced G/GEJ cancer, objective response was 9%–17% with irinotecan monotherapy and 9%–22% with irinotecan-containing doublet treatment, median PFS was 2.3–3.4 and 2.4–3.8 months and median OS was 8.4–10.3 and 8.3–10.7 months, respectively.^20–23^ Anti-PD-1 antibody monotherapy for advanced G/GEJ cancer showed limited clinical activity compared with chemotherapy.^8 24^ In KEYNOTE-061 trial, which was a phase III trial with pembrolizumab compared with paclitaxel as second-line therapy in patients with advanced or metastatic G/GEJ adenocarcinoma, ORR was 16% (95% CI 11% to 22%) with pembrolizumab group and 14% (95% CI 9% to 19%) with paclitaxel group with a PD-L1 CPS of 1 or higher, median PFS was 1.5 and 4.1 months, median OS was 9.1 and 8.3 months, and the estimated proportion of patients surviving at 1 year was 40% and 27%, respectively.^8^ By contrast, confirmed objective response was 27.6% in FAS and 30.2% in evaluable patients in this study. Similar to previous reports of immunotherapies combined with chemotherapy, early and durable response were observed. Median PFS was numerically longer compared with previous reports of irinotecan monotherapy.^22^ While median OS was NR within a median follow-up of 10.5 months. Notably, objective response and PFS with HX008 combined with irinotecan was independent of tumor PD-L1 expression, whereas OS benefit tends to be more apparent in patients with PD-L1 positive tumors (CPS ≥1). The correlation of clinical benefit with tumor PD-L1 expression was indeterminate in advanced G/GEJ cancer. Antitumor response with nivolumab was independent of PD-L1 status, while clinically meaningful efficacy with pembrolizumab was observed in CPS ≥10.^25^ Overall, these results were comparable with reports of ICIs combination treatment in patients with advanced or recurrent G/GEJ cancer,^26–28^ and were similar to those previously reported for ramucirumab plus paclitaxel,^10^ which suggest that HX008 plus irinotecan may be a promising therapeutic option as second-line therapy.

AEs associated with HX008 and irinotecan were in general of mild severity and manageable. The main AEs were myelosuppression caused possibly by irinotecan. The hematologic toxicities were similar to previous reports with FOLFIRI as treatment for advanced GC.^29^ In irinotecan monotherapy as third-line or later treatment in advanced GC, grade 3 or 4 neutropenia, anemia and thrombocytopenia appeared in 19.2%, 19.2% and 1.4% respectively,^30^ the results were basically consistent with our trials. Most of the immune-related AEs were grade 1 or 2. Previous studies have reported that the most common immune-related AEs associated were fatigue, pruritus, rash, hypothyroidism, proteinuria and decreased appetite.^31^ In our study, all these AEs were observed and no unexpected AEs or new safety signals were identified.

We would also like to acknowledge that its single-arm nature with the limited number of patients limited our ability to compare the findings directly with available therapies for this patient population. On the other hand, other biomarkers, including Epstein-Barr virus positivity or microsatellite instability, were not detected in our trial, which may be a limitation of this report.

In conclusion, these results suggest that HX008 injection in combination with irinotecan in patients with unresectable advanced or recurrent G/GEJ cancer may be a potential second-line therapeutic option with a manageable safety profile and encouraging efficacy. A randomized phase III trial of HX008 plus irinotecan vs placebo plus irinotecan will be initiated to further evaluate the combination as second-line treatment in advanced G/GEJ cancer.
