# Supplementary material for: HX008, an anti-PD1 antibody, plus irinotecan as second-line treatment for advanced gastric or gastroesophageal junction cancer: a multicenter, single-arm phase II trial
Source: J Immunother Cancer. 2020 Oct 15;8(2):e001279. doi: 10.1136/jitc-2020-001279 (PMC7566427; doi:10.1136/jitc-2020-001279)

Supplementary Figure 1. Patient disposition

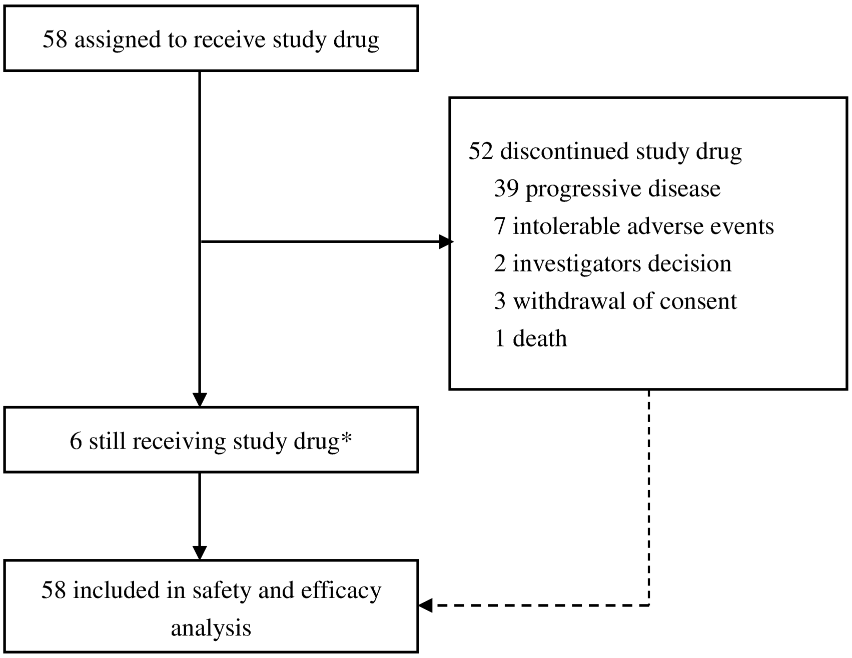

*At analysis cutoff date (May 11, 2020)

Song Y, *et al. J Immunother Cancer* 2020; 8:e001279. doi: 10.1136/jitc-2020-001279

Supplementary Figure 2. Kaplan-Meier estimates of PFS (A) and OS (B) according to PD-L1 expression.

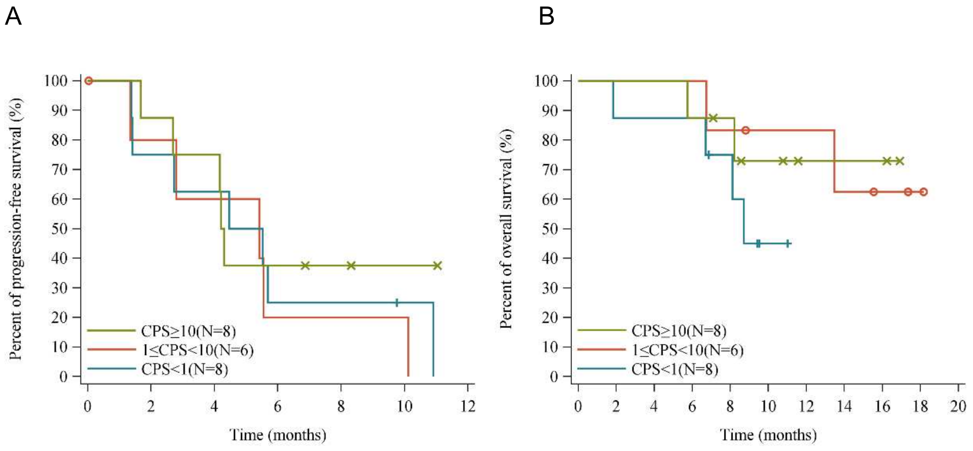

Supplement: Supplementary data [file jitc-2020-001279supp001.pdf]
